# Identifying accelerometer nonwear and wear time in older adults

**DOI:** 10.1186/1479-5868-10-120

**Published:** 2013-10-25

**Authors:** Brent Hutto, Virginia J Howard, Steven N Blair, Natalie Colabianchi, John E Vena, David Rhodes, Steven P Hooker

**Affiliations:** 1 Prevention Research Center, Arnold School of Public Health, University of South Carolina, 921 Assembly Street, Columbia, SC 29208, USA; 2 Department of Epidemiology, School of Public Health, University of Alabama at Birmingham, Ryals Bldg 210 F, 1665 University Blvd, Birmingham, AL 35294-0022, USA; 3 Departments of Exercise Science and Epidemiology and Biostatistics, Arnold School of Public Health, University of South Carolina, 921 Assembly Street, Columbia, SC 29208, USA; 4 Institute for Social Research, University of Michigan, P.O. Box 2346, 426 Thompson Street, Ann Arbor, MI 48106, USA; 5 Department of Epidemiology and Biostatistics, College of Public Health, University of Georgia, B.S. Miller Hall Room 105, 101 Buck Road, Athens, GA 30602, USA; 6 Department of Biostatistics, School of Public Health, University of Alabama at Birmingham, 912 Building, Suite 200, 1720 2nd Ave. South, Birmingham, AL 35294-0022, USA; 7 Exercise and Wellness Program, School of Nutrition and Health Promotion, Arizona State University, 500 North Third Street, MC 3020, Phoenix, AZ 85004, USA

**Keywords:** Activity monitor, Physical activity assessment, Nonwear classification, Sedentary behavior, Aging

## Abstract

**Background:**

Five accelerometer-derived methods of identifying nonwear and wear time were compared with a self-report criterion in adults ≥ 56 years of age.

**Methods:**

Two hundred participants who reported wearing an Actical™ activity monitor for four to seven consecutive days and provided complete daily log sheet data (i.e., the criterion) were included. Four variables were obtained from log sheets: 1) dates the device was worn; 2) time(s) the participant put the device on each day; 3) time(s) the participant removed the device each day; and 4) duration of self-reported nonwear each day. Estimates of wear and nonwear time using 60, 90, 120, 150 and 180 minutes of consecutive zeroes were compared to estimates derived from log sheets.

**Results:**

Compared with the log sheet, mean daily wear time varied from -84, -43, -24, -14 and -8 min/day for the 60-min, 90-min, 120-min, 150-min and 180-min algorithms, respectively. Daily log sheets indicated 8.5 nonwear bouts per week with 120-min, 150-min and 180-min algorithms estimating 8.2-8.9 nonwear bouts per week. The 60-min and 90-min methods substantially overestimated number of nonwear bouts per week and underestimated time spent in sedentary behavior. Sensitivity (number of compliant days correctly identified as compliant) improved with increasing minutes of consecutive zero counts and stabilized at the 120-min algorithm. The proportion of wear time being sedentary and absolute and proportion of time spent in physical activity of varying intensities were nearly identical for each method.

**Conclusions:**

Utilization of at least 120 minutes of consecutive zero counts will provide dependable population-based estimates of wear and nonwear time, and time spent being sedentary and active in older adults wearing the Actical™ activity monitor.

## Background

The utilization of accelerometers to objectively monitor physical activity-related movement has become widespread over the past 15 years. Despite some limitations, these devices are well-suited to capture data associated with the most common form of locomotion in adults ≥ 50 years of age, namely walking. Several studies have been conducted to develop algorithms and activity count cut-points to aid in the differentiation between light, moderate and vigorous intensity physical activity in persons of varying ages wearing accelerometers [1-6]. Such advancements have increased the use of accelerometers in population-based studies to better understand the prevalence of physical activity [7,8] and explore the association between physical activity components and multiple health outcomes [9-11].

A focus on time spent being sedentary has emerged with evidence indicating that sedentary behavior is significantly associated with several health-related factors independent of physical activity level [12,13]. Many researchers view accelerometry as a suitable method for measuring sedentary time in adults [12,14,15]. However, a challenge remains as both time spent in sedentary behavior and simply not wearing the device will result in an activity count of zero being recorded. In a 2005 study, a participant was considered not to be wearing the device if 20 consecutive minutes of zero activity counts were recorded [16]. This method was later modified with a commonly applied wear-time estimation algorithm using a string of 60 consecutive zeros, sometimes with minor allowances for interruptions to denote nonwear [8,17]. However, recent studies have shown that the estimated wear time can be sensitive to the specific number of consecutive zero counts defined as nonwear. To avoid large underestimates of wear time, these comparison studies have recommended longer strings of zeroes (e.g., 90-120 minutes) than were commonly employed in the past [15,18-22].

Automated wear-time estimation algorithms are particularly desirable for large-scale studies (e.g., prospective cohort studies), but they are not yet standardized [16,20]. Accuracy needs to be established because misclassifying sedentary behavior as nonwear could result in unnecessary loss of valuable data. In addition, analyses focused on sedentary behavior will be severely weakened by the underestimation of time spent being sedentary due to overestimation of nonwear time. Furthermore, inaccurate estimation of nonwear time directly impacts calculated wear time which could result in invalid estimates of accelerometer-derived physical activity measures [19,23].

Previous studies of automated wear-time estimation algorithms have only been conducted within restricted populations and a limited type of instruments [18-21,24]. Only two of the published studies to date have focused solely on an older adult population, neither of which employed the Actical™ activity monitor [24,25]. One prior study included the Actical™ activity monitor, but this was done with a younger adult population [22]. The purpose of this study was to compare five different accelerometer-derived methods of identifying nonwear and wear time (using the Actical™ activity monitor) with a daily log sheet criterion in a general population sample of adults ≥ 56 years of age. This was done to determine the length of consecutive-zero strings resulting in the most accurate nonwear and wear time estimates in population-based studies investigating physical activity, sedentary behavior, and health outcomes in older adults.

## Methods

### Study population

The REasons for Geographic and Racial Differences in Stroke (REGARDS) Study comprises a general population sample residing in the United States designed to prospectively examine racial and regional disparities in stroke risk and mortality. Detailed design and methods for the REGARDS study, managed by the University of Alabama at Birmingham (UAB), have been described elsewhere [26]. Overall, 30,239 black and white participants, aged 45 and older, oversampled from the southeastern stroke belt and buckle, were recruited in 2003–2007 from commercially available lists, and screened during a phone interview to determine eligibility. Following verbal consent, using a computer-assisted telephone interview (CATI), trained interviewers obtained demographic information and medical history. A physical examination was conducted in-person 3–4 weeks after the telephone interview, and written informed consent was obtained. The telephone response rate was 33% and cooperation rate was 49%, similar to other cohort studies [27]. Participants are contacted by telephone every six months for cognitive assessments and surveillance of medical events.

### Accelerometer ancillary study

An ancillary study was commenced in August 2009 to collect an objective measure of physical activity. A total of 1,150 Actical™ activity monitors (Mini Mitter Respironics, Inc., Bend, OR) were available to be cycled through the participants. During routine six-month follow-up telephone calls by the CATI unit, if an Actical™ activity monitor was available, prospective participants responded to an eligibility question asking whether or not on a typical day they were able to go outside of their house and walk. If the response was affirmative, the participant was provided with a brief explanation of the purpose of the study and asked if he/she would be willing to wear the accelerometer for seven consecutive days and complete a daily log sheet. As of May 2013, over 12,000 participants had agreed to join the ancillary study. Prospective participants were told it did not matter what their current level of physical activity was, to expect the device to arrive in the mail within the next week, and to start wearing the device immediately the day after receipt. If the participant agreed to wear the device (i.e., verbal consent was provided), the CATI unit notified UAB staff responsible for implementing the accelerometer protocol.

Once notified, staff initialized the Actical™, secured it to an adjustable nylon belt, and mailed it to the participant along with a cover letter, written and pictorial wear instructions, daily log sheet, protocol check list, and pre-addressed and postage-paid return envelope. Participants were instructed to start wearing the device the day after they first received it, remove at bedtime and reattach upon awakening, position the device over the right iliac crest, make sure the belt was snug around the waist, complete the daily log sheet with start date and time the device was put on and taken off each day, and return the device immediately after completing the seven day protocol. A toll-free telephone number was provided if the participant had questions or concerns. Reminder post cards were mailed two to three days following the initial mailing of the device to encourage compliance, and staff initiated follow-up postcards and telephone calls if the device was not received back at UAB within 45 or 65 days, respectively.

The Actical™ activity monitor used for this study is water resistant, lightweight (17 g), small (2.8 × 2.7 × 1.0 cm^3^), and has a data storage capacity of 64,800 data points that will saturate after 44 days of continuous measurement using 1-min recording intervals (epochs). The monitor was initialized and any available data downloaded before each mailing using a serial port computer interface. The Actical™ uses a single internal “omnidirectional” accelerometer that senses motion in all directions, but is most sensitive within a single plane. It detects low frequency (0.5 to 3.2 Hz) G-forces (0.05 to 2.0 Hz) common to human movement and generates an analog voltage signal that is filtered and amplified before being digitized by an A-to-D converter at 32 Hz. In this study, the digitized values were summed over 1-minute epochs. The actual numbers stored by the Actical™ are proportional to the magnitude and duration of the sensed accelerations. Based on our [2] and other’s [1,15] work, activity count cut-points of 100 counts per minute (cpm) and 1065 cpm were applied to differentiate between being sedentary and light intensity physical activity and light and moderate or higher intensity physical activity, respectively.

Institutional Review Boards at the Arizona State University, University of South Carolina, University of Georgia and University of Alabama at Birmingham approved the study methods which, due to previous written consent provided for the parent study and the low participant burden of the ancillary study, required only verbal consent from the participant at the time of the recruitment phone call.

### Inclusion/exclusion criteria

Each participant was asked to complete a one-page daily log sheet with three elements: the date on which the Actical™ was first worn, the time(s) on and off for each of the following seven days including multiple on and off times for any given day, and any comments the participant believed would clarify their physical activity data as recorded by the monitor. While nearly all log sheets include the date of the first day of wear, they vary in completeness of additional day-by-day information that is requested. To be used in the current analysis as a criterion, a log sheet also had to provide daily on/off times for at least four of the seven days immediately following the beginning of wear.

Although others have cautioned against using self-report of exact wearing practices (i.e., daily log sheets) to estimate wear time [19,28], we were fortunate to have several hundred meticulously completed daily log sheets from which to select. Some log sheets contained more than one time of day when the participant put on and/or removed the accelerometer with notations of why this was done. The careful selection of such detailed daily log sheets greatly increased our confidence in utilizing them as a criterion measure of wear and nonwear time. This approach is not without precedent as two recently published studies that investigated the use of automated estimates of accelerometer nonwear and wear time also relied on self-reported information provided via daily activity logs as a criterion of whether or not participants were wearing an accelerometer [20,22]. Others have also used well kept daily logs to verify short nonwear intervals during waking hours [24,25].

A randomized list of all participants was created and used to select log sheets for examination to eliminate those which were not fully completed or had illegible entries. An initial sample of 100 log sheets having daily on/off times for four or more days was examined and found to contain almost entirely participants who reported wearing the Actical™ for seven days. The second half of the desired sample of 200 log sheets was selected with modified criteria specifying only four, five or six days of reported Actical™ wear so we could examine both compliant and noncompliant days. However, very few legible and complete log sheets with only four or five self-reported compliant days were present in the database. Thus, the majority of non-seven-day participants have six days of reported wear and the total number of noncompliant days in the sample of 200 is relatively small (N = 63).

### Estimation of nonwear time

Data reduction was performed (manually) on each log sheet to produce a dataset with daily wear and nonwear periods as reported by the participant. Each log sheet entry is recorded in the participant’s local time zone and daylight-savings setting. However the Actical™ clock stays set to Central time zone and the daylight-savings setting in effect at the time of its last battery change. So an additional (manual) reconciliation was required to correct the Acitcal™ and log sheet times. In a few cases, the time zone difference could not be determined with certainty and those log sheets were discarded and replaced with others drawn from the original randomized list of log sheets. There were also a few cases in which self-reported wear dates did not correspond with the presence of valid data in the Actical™ (AWC) file due to memory overflow or device failure. Log sheets from these participants were also replaced with others drawn from the original randomized list.

For each of the seven consecutive days beginning on the initial wear date, three types of information were abstracted from the completed daily log sheet: 1) date (day-month-year) for that day; 2) one or more time(s) at which the participant put on the Actical™; 3) time(s) at which the participant removed the Actical™. These items were transcribed along with the participant ID into a dataset. The five methods of estimating nonwear time were similar except for the parameter specifying the number of consecutive zeros to be treated as “nonwear”. Based on previous studies [18-21,24], we compared estimates of nonwear using 60, 90, 120, 150 and 180 minutes of consecutive zeroes. The seven days of data (midnight on Day 1 through 11:59 PM on Day 7) were processed as a continuous vector of counts that are 1-minute epochs so that zero-count strings continue across the daily midnight boundary. Continuous nonwear estimation rather than stopping and restarting the algorithm daily at midnight has been identified as important to avoid misclassification of wear or nonwear periods in cases when the actual wear stops and nonwear starts after 11:00 pm [18]. In this process, each epoch is categorized as nonwear, sedentary, or light, moderate or vigorous intensity physical activity resulting in a dataset with 10,080 records per individual, each containing a date-time value, an activity count, and indicator variables with a value of one for that epoch’s activity category and zero otherwise. Later analytic procedures organized the string of epochs into days and summed the minutes spent in each category of physical activity or nonwear on a daily basis.

All daily wear-time estimates for the five accelerometer methods were compared to the number of minutes of wear derived from the on/off times and any self-reported nonwear periods on the log sheet. Time-of-wear difference variables were obtained by subtracting log sheet nonwear duration from the number of minutes of wear extracted for each of the five accelerometer nonwear estimation methods.

A common protocol for assuring validity is to exclude days on which the accelerometer is worn less than some minimum time, often 10 hours/day [8]. In 7-day physical activity monitoring it is also common to exclude the data from any participant who fails to provide four or more such “compliant days” [16]. An additional criterion of at least one weekend day is sometimes applied but REGARDS-PA specifies that any four or more days in a seven-day period will be used. According to daily log sheet data, all participants in our select sample met the 10 hours/day threshold for four to seven days. However, estimated wear time from the various methods occasionally resulted in one or more self-reported compliant days misidentified as noncompliant or self-reported noncompliant days misidentified as compliant based on a 10 hour/day threshold. The proportion of false-noncompliant and false-compliant days misidentified by each method is an important performance metric for comparing methods.

## Results

Participants for this study were relatively evenly divided by sex (48% female) and residential location (52% living in the stroke belt), majority white (72%), overweight (body mass index = 29.2 ± 6.4 kg/m^2^), well-educated (72% some college/college graduate), and aged between 56 and 74 years (63.5 ± 8.3 years). Other than a slightly higher proportion of white participants and those living outside of the stroke belt, these features closely reflect those of the entire REGARDS cohort. Demographic characteristics are listed in Table 1.

**Table 1:** **Characteristics of participants (N = 200)**^***a***^

	
Female: N (%)	115 (48%)
Race: N (%)	
White	144 (72%)
Black	56 (28%)
Age (years): Mean ± SD	63.5 ± 8.3
BMI (kg/m^2^): Mean ± SD	29.2 ± 6.4
Education level: N (%)	
Less than high school	10 (5%)
High school graduate	44 (22%)
Some college	61 (30.5%)
College graduate and above	85 (42.5%)
Annual income: N (%)	
< $20 K	19 (9.5%)
$20-$34 K	46 (23%)
$35-$74 K	78 (39%)
$75 K+	38 (19%)
Refused	19 (9,5%)
Relationship status	
Married	132 (66%)
Divorced	19 (9.5%)
Single/Other/Widowed	49 (24.5%)
Heart Disease^*b*^: N (%)	25 (12.5%)
Hypertension^*c*^: N (%)	96 (48%)
Self-reported stroke^*d*^: N (%)	9 (4.5%)
Diabetes^*e*^: N (%)	33 (16.5%)
Region: N (%)	
Belt^*f*^	71 (35.5%)
Buckle^*g*^	33 (16.5%)
Non-Belt^*h*^	96 (48%)

*Abbreviations:SD* standard deviation, *BMI* body mass index.

^*a*^Demographic data collected at baseline when participants were initially enrolled in parent study.

^*b*^Heart disease includes self-reported myocardial infarction, coronary artery bypass graft, bypass, angioplasty, stent, or evidence of myocardial infarct via electrocardiogram; 4 participants with missing data.

^*c*^Hypertension includes systolic blood pressure ≥140 mmHg, diastolic blood pressure ≥90 mmHg, or self-reported current medication use to control blood pressure.

^*d*^2 participants with missing data.

^*e*^Diabetes includes fasting glucose ≥126 mg/dl, non-fasting glucose ≥200 mg/dl, or self-reported pills or insulin.

^*f*^Buckle: coastal plain region of North Carolina, South Carolina, and Georgia.

^*g*^Belt: remainder of North Carolina, South Carolina, and Georgia plus Alabama, Mississippi, Tennessee, Arkansas, and Louisiana.

^*h*^Non-belt: other 40 contiguous states.

According to the criterion daily log sheets, participants wore the device an average of 851 ± 159 minutes, or just over 14 hours per day during the accelerometer protocol. Table 2 displays the estimated number of compliant days, mean daily wear time (min/day), mean weekly wear time (min/week), sensitivity and specificity for compliant days, and number of nonwear bouts per week derived for each of the five algorithms. Compared with the daily log sheet, mean daily wear time varied from -84, -43, -24, -14 and -8 min/day for the 60-min, 90-min, 120-min, 150-min, and 180-min algorithms, respectively. The absolute difference in daily wear time was nearly identical across the 120-min, 150-min and 180-min algorithms and less than those for the 60-min and 90-min methods. However, the large standard deviations of 140-152 min/day (corresponding RMSE = 156-189 min/day) indicate substantial individual variability and potential error for this variable inherent with all algorithms.

**Table 2:** **Comparison of daily log sheet and automated algorithms**^***a ***^**in classifying estimated number of compliant days**^***b***^**, daily wear time, and bouts of nonwear**

	**Daily log Sheet**	**60-min**	**90-min**	**120-min**	**150-min**	**180-min**
Wear time (min/day); Mean ± SD	851 ± 159	767 ± 199	808 ± 192	827 ± 190	837 ± 189	843 ± 188
Absolute difference wear time (min/day);		113 ± 152	85 ± 142	74 ± 140	71 ± 140	70 ± 140
Mean ± SD						
Root-mean-square difference (min/day)		189	166	158	157	156
Compliant days; Mean ± SD	6.7 ± 0.6	6.0 ± 1.4	6.3 ± 1.1	6.4 ± 1.0	6.4 ± 1.0	6.5 ± 0.9
Compliant days misidentified		171 (13%)	96 (7%)	77 (6%)	68 (5%)	62 (5%)
as noncompliant						
Noncompliant days misidentified		13 (20%)	16 (25%)	17 (27%)	19 (30%)	21 (33%)
as compliant						
Sensitivity (Compliant days correctly		87.2%	92.8%	94.2%	94.9%	95.4%
identified as compliant)						
Specificity (Noncompliant days correctly		79.4%	74.6%	73.0%	69.8%	66.7%
identified as noncompliant)						
Nonwear bouts/Week; Mean ± SD	8.5 ± 1.4	14.3 ± 5.6	10.3 ± 2.7	8.9 ± 1.5	8.4 ± 1.1	8.2 ± 0.8

*Abbreviations:min* minute, *SD* standard deviation, *PA* physical activity, *MVPA* moderate to vigorous intensity physical activity.

^*a*^The algorithms define nonwear periods as continuous non-movement (i.e., 0 cpm) of the specified duration.

^*b*^Compliant day is defined as the participant wearing the accelerometer for ≥10 hours/day.

The daily log sheets indicated 8.5 nonwear bouts per week with the 120-min, 150-min and 180-min algorithms performing well with estimates ranging from 8.2-8.9 nonwear bouts per week. The 60-min and 90-min methods substantially overestimated the number of nonwear bouts per week with values of 10.3 and 14.3 nonwear bouts per week, respectively.

Sensitivity (i.e., correct identification of compliant days as compliant) improved with increasing minutes of consecutive zero counts in the algorithm with the 120-min, 150-min and 180-min algorithms having relatively stable rates of 94.2%-95.8%. As expected, specificity (i.e., correct identification of noncompliant days as noncompliant) was highest for the 60-min and 90-min algorithms. Among the other three methods, the 120-min algorithm (73.0%) outperformed the 150-min (69.8%) and 180-min (66.7%) methods with respect to correctly identifying noncompliant days.

Despite differences in mean daily wear time between the log sheet criterion and each algorithm, the proportion of wear time being sedentary or involved with moderate to vigorous physical activity (MVPA) and the absolute time spent in light physical activity or MVPA was nearly identical for each method (Table 3). The data demonstrated this sample was primarily sedentary during awake hours (75-77% of total wear time) and engaged in a very limited amount of MVPA (approximately 14 minutes per day), although they accumulated nearly three hours per day of light intensity physical activity.

**Table 3:** **Comparison of automated algorithms**^***a ***^**in estimating daily time spent being sedentary and physically active at various intensities**

	**60-min**	**90-min**	**120-min**	**150-min**	**180-min**
Sedentary time (min/day)^*b*^; Mean ± SD	618 ± 81	649 ± 88	667 ± 97	675 ± 98	679 ± 101
Sedentary time (% of total wear time)	75 ± 10	77 ± 10	77 ± 10	77 ± 10	77 ± 10
Light Intensity PA (min/day)^*b*^; Mean ± SD	190 ± 76	187 ± 77	186 ± 77	185 ± 76	185 ± 77
MVPA (min/day)^*b*^; Mean ± SD	14 ± 19	14 ± 19	14 ± 19	14 ± 19	14 ± 19
MVPA Time (% of total wear time)	1.7 ± 2.1	1.6 ± 2.1	1.6 ± 2.0	1.6 ± 2.0	1.6 ± 2.0

*Abbreviations:min* minute, *SD* standard deviation, *PA* physical activity, *MVPA* moderate to vigorous intensity physical activity.

^*a*^The algorithms define nonwear periods as continuous non-movement (i.e., 0 cpm) of the specified duration.

^*b*^Accelerometer cut-points for inactive time = 0-100 cpm, light intensity PA = 101-1064 cpm and MVPA =1065 cpm or higher.

## Discussion

This study compared accelerometer-derived methods of distinguishing nonwear from time spent being sedentary with a daily log sheet criterion. To our knowledge, this is the first such study to employ the Actical™ activity monitor with a specific sample of older adults. These comparisons are essential to ascertain the most accurate method of determining wear and nonwear time in *population-based* studies investigating physical activity, sedentary behavior, and health outcomes in older adults. In particular, incorrect interpretation of extended periods (i.e., one hour or longer) of sedentary behavior as being nonwear contributes directly to erroneous sedentary time estimates and indirectly to misidentifying compliant days as noncompliant.

Our results indicated the 120-min, 150-min, and 180-min algorithms provided nearly equivalent results in estimated wear and nonwear time, number of compliant days, and number of nonwear bouts with each closely aligned with the log sheet criterion. These algorithms also displayed similar high levels of correctly identifying compliant days (i.e., sensitivity) with the 120-min method having a slight edge with respect to correctly identifying noncompliant days. Thus, each of these methods would be strongly favored over the 60-min and 90-min methods to most accurately identify wear and nonwear time in persons 50 years of age or older. A prior study using the Actical™ with a younger adult population (mean age = 42 ± 12 years) reported a threshold of 180 consecutive minutes with a 1-min allowance before or after the bout yielded the most accurate determination of accelerometer nonwear compared with a 60-min method [22]. Findings of the current study do not refute the use of either a 150-min or 180-min consecutive zero nonwear algorithm. However, our results do indicate a relative stabilization in estimated wear time, compliant days, and nonwear bouts, and sensitivity among the 120-min, 150-min and 180-min thresholds. Song et al. [24] reported a similar stabilization of results in midlife and older adults with knee osteoarthritis near the 90-min to 120-min threshold out to 300 minutes of consecutive zeros. It is especially worth noting that our and others’ findings indicate that older adults, particularly those who are quite sedentary, can wear an accelerometer for extensive periods of time without accumulating any counts.

According to the daily log sheet criterion, all 200 participants provided four or more compliant days, with 188 having six or seven compliant days. However, the 60-min, 90-min and 120-min methods resulted in fewer than four compliant days for some subjects. In many studies, data from these participants would be excluded resulting in an even greater error in estimates due to misidentification of compliant days as noncompliant. Although the 60-min and 90-min algorithms had greater specificity, researchers are generally more concerned about having compliant days misidentified (i.e., lower sensitivity). The two shorter threshold methods also substantially underestimated daily wear time and sedentary time. Thus, as stated above, these two intervals do not seem as appropriate for use with older adults when employing the Actical™ activity monitor at the waist.

Previous studies using different methodologies have determined that a longer than 60-min window of zero counts is required to improve the accuracy of wear and nonwear time estimation [18,19,24,25], and our results support those findings. In bariatric patients wearing a Stepwatch activity monitor on the ankle, King et al. [19] concluded that a 120-min interval yielded the most reasonable estimates of daily wear and nonwear periods. The investigators stated that the 150-min interval resulted in too high an estimate of the number of people who never removed the device during the day. However, the number of compliant days determined with the 120-min and 150-min intervals did not differ significantly. Interestingly, our results revealed algorithms with 120-min, 150-min and 180-min of consecutive zeros provided accurate and similar estimates of the number of nonwear bouts per week, thereby correctly identifying when the device was removed and ultimately contributing to nearly precise estimates of the number of compliant days.

Choi et al. [18] conducted a validation study of nonwear algorithms by having participants age 39 ± 13 years wear an Actigraph GT1M accelerometer placed on the waist during a strictly monitored 24-hour protocol in a room calorimeter. Their results showed a 90-min zero-count algorithm improved accuracy of wear and nonwear time misclassification compared with a 60-min algorithm. However, in addition to the 90-min window with zero counts, the method also required a new component of an upstream and downstream 30-min consecutive non-zero count window with the allowance of 2-min non-zero window for detection of artifactual movement (sometimes referred to as spurious counts). Other studies have also included allowances for artifactual movement in their automated wear algorithms [22,24]. Such an allowance may be necessary in datasets including overnight (i.e., 24 hour/day) accelerometer wear but, absent that consideration, allowing non-zero counts during estimated nonwear periods will only serve to increase misclassification of wear as nonwear.

As Choi et al. [25] recently point out, among older adults the time spent in sedentary behaviors is much greater than relatively short periods when a monitor is removed. Two studies comparing the estimation of nonwear and sedentary time using 60-min [24] and 180-min [22] algorithms with and without allowances for limited interruptions in consecutive zero counts noted only very slight improvements with the algorithm allowing interruptions. Another study investigating the most accurate method to differentiate between sedentary behavior and nonwear time noted a difference of only 4-5 minutes per day when comparing algorithms with and without ≤2-min allowances for interruptions in consecutive zero counts [20]. Therefore, in the current study, only truly continuous zero-count strings with no allowance for artifactual movement were considered nonwear. Of course, if investigators have access to software with algorithms allowing for spurious counts, they should select such methods if suitable for the device being used and their study population. However, we propose that unless movement of the accelerometer while not being worn is a frequent occurrence, which seems unlikely for a typical participant, accounting for this possibility with “artifactual” allowances may not be worth increasing the risk of sedentary periods being misidentified as nonwear.

Any over- or underestimation of nonwear time could significantly impact the estimation of sedentary time. As observed in Table 3, although the absolute time spent in sedentary behavior was underestimated by the 90-min and especially the 60-min method, the proportion of total time spent being sedentary and absolute and proportion of time spent in light intensity physical activity and MVPA were nearly identical between each of the five algorithms. One other study also indicated time spent in MVPA was unaffected by the consecutive zero length of the nonwear algorithm [24]. However, due to greater accuracy in estimating other variables and higher sensitivity, our findings support the use of an algorithm with at least 120 minutes of consecutive zeros in populations of older adults wearing the Actical™ on the waist, and this will allow confidence in the resulting estimates of both time spent being sedentary and physically active.

The appropriate number of participants to include in a study such as this is unknown. Our sample size was significantly greater than that of four previous studies [18,20,22,25] and substantially less than in another [12]. Yet, it is highly unlikely the results would have varied with a larger number of participants. Use of the self-report log as a criterion is not without limitations. However, this method has been used as a criterion for accelerometer wear and nonwear previously and, as noted by authors of those studies, was unlikely to favor any particular algorithm and adequate for comparison purposes [20] and is a viable alternative to direct observation which is not practical with large samples [21,22]. We were fortunate to have several hundred participants to choose from and only included a subsample with very detailed log information indicating days of wear and times of daily wear and nonwear. This greatly increased our confidence in the daily log sheet outcomes. Our sample was comprised of older adults and is not fully representative of all age groups. However, our participants were selected from a national sample of older adults comprised of nearly equal proportions of men and women and a substantial proportion of blacks. The 200 participants included in the analysis also had nearly identical values for mean weekly wear time (851 min vs 884 min), mean proportion of wear time spent being sedentary (77.0% vs 77.5%), and mean proportion of wear time spent in MVPA (1.6% vs 1.5%) as the overall sample included in the REGARDS-PA ancillary study to date (unpublished results). Still, it is possible that participants willing to enroll in a longitudinal cohort study are more compliant than the general population in terms of completing a daily log sheet and wearing the Actical™ as requested. Thus, generalizability is uncertain, and additional studies with populations varying in age, sex, race/ethnicity and functional status are recommended. Further investigation and refinement of automated nonwear algorithms with different activity monitors will also be needed as technology improves.

It is not possible to determine a minimum duration of zero counts that best differentiates between periods of wear from nonwear for all participants [3,19]. Indeed, any duration used will result in some misclassification. As noted by the large standard deviations in daily wear time estimates for our sample (Table 2) and in other studies [21,22], there is wide individual variation and potential error with these nonwear algorithms. Thus, we strongly caution the use of these methods and interpretation of data with relatively small sample sizes as might be included in clinical and/or intervention trials. Nonetheless, the results of the current study indicate that, when wearing an Actical™ activity monitor at the waist, utilization of at least 120 minutes of consecutive zero counts will provide dependable *population-based* estimates of wear and nonwear time, and time spent being sedentary and physically active at varying intensities in persons ≥56 years of age. As previously suggested [18], the proposed automated estimation algorithm will be especially useful in large-scale population studies in which assessment of physical activity and sedentary behavior are linked to longitudinal health risks and disease outcomes.

## Competing interests

The authors declare they have no competing interests.

## Authors’ contributions

VJH and DR coordinated the acquisition of data. BH conceived and designed the study, conducted data analysis, interpreted the data, and drafted the methods and results sections of the manuscript. SPH had general supervision of the study, helped with interpretation of the data, and drafted the introduction and discussion sections of the manuscript. All authors critically read the manuscript for intellectual content and approved the final version to be published.
